# Supplementary material for: Evaluation of Reference Genes for Real-Time Quantitative PCR Analysis in Tissues from Bumble Bees (Bombus Terrestris) of Different Lines
Source: Int J Mol Sci. 2022 Nov 19;23(22):14371. doi: 10.3390/ijms232214371 (PMC9692494; doi:10.3390/ijms232214371)
Supplement: Supplementary file 1 [file ijms-23-14371-s001.zip › ijms-1997681-supplementary.pdf]

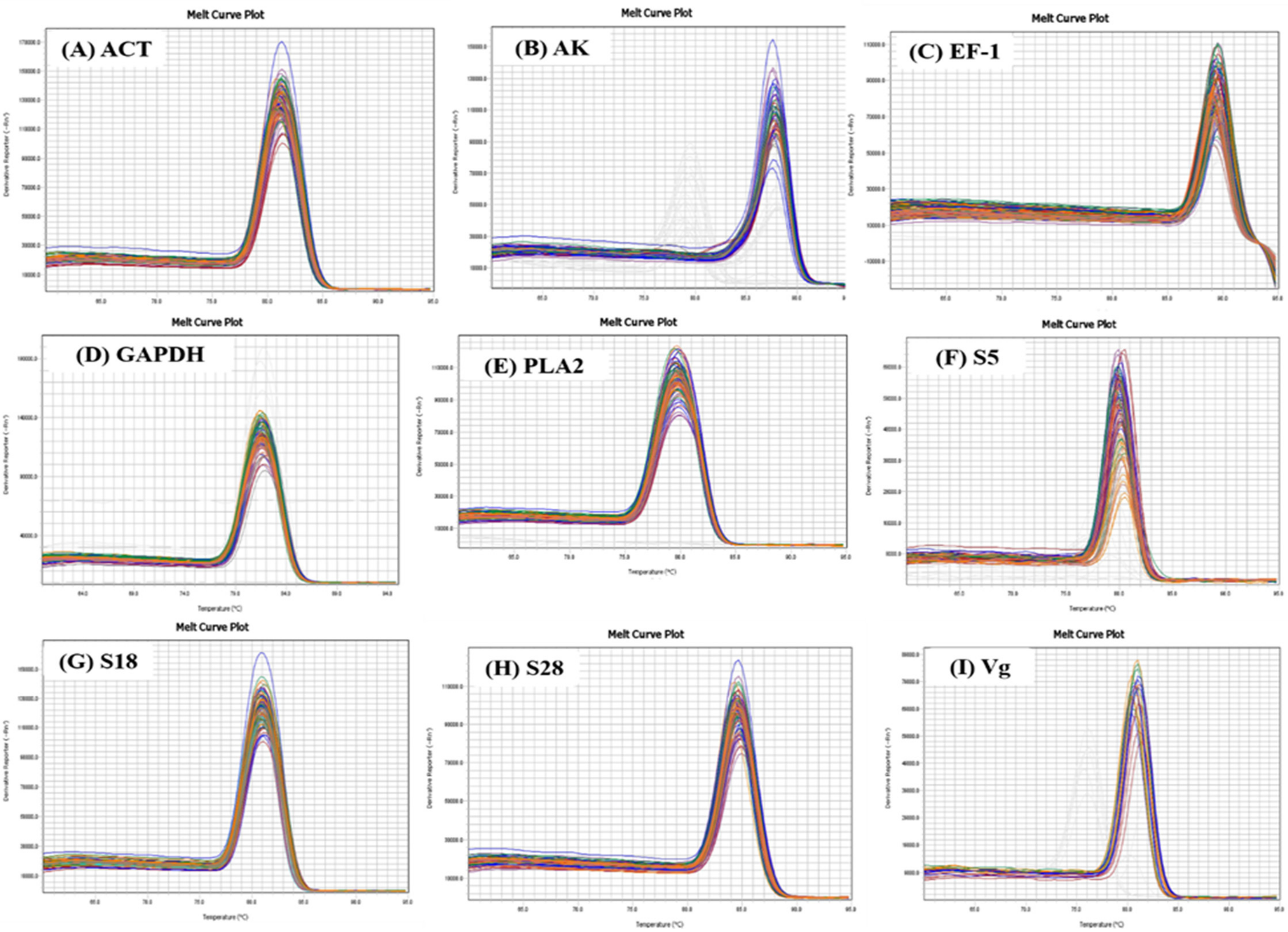

Figure S1. Melting curve analysis of eight reference genes (ACT, AK, EF-1, GAPDH, PLA2, S5, S18, S28, and one Target gene Vg.

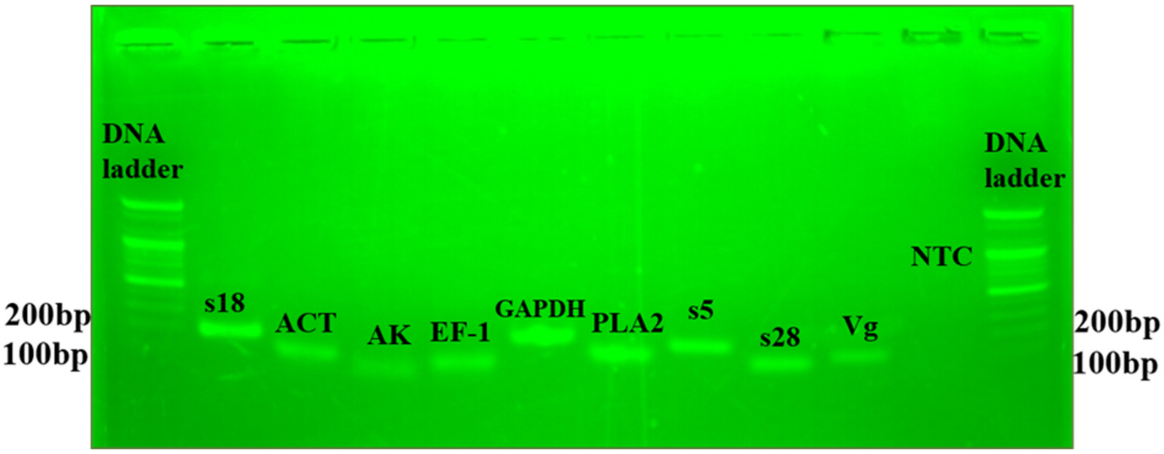

Figure S2. 1% Agarose gel electrophoresis Analysis of eight gene primer specificity
